# Prediction of liquid–liquid phase separating proteins using machine learning

**DOI:** 10.1186/s12859-022-04599-w

**Published:** 2022-02-15

**Authors:** Xiaoquan Chu, Tanlin Sun, Qian Li, Youjun Xu, Zhuqing Zhang, Luhua Lai, Jianfeng Pei

**Affiliations:** 1 Center for Quantitative Biology, Academy for Advanced Interdisciplinary Studies, Peking University, Beijing, 100871 China; 2 College of Information and Electrical Engineering, China Agricultural University, Beijing, 100083 China; 3 College of Life Sciences, University of Chinese Academy of Sciences, Beijing, 100049 China; 4 Beijing National Laboratory for Molecular Science, State Key Laboratory for Structural Chemistry of Unstable and Stable Species, College of Chemistry and Molecular Engineering, Peking University, Beijing, 100871 China; 5 Peking-Tsinghua Center for Life Sciences, Peking University, Beijing, 100871 China

**Keywords:** Liquid–liquid phase separation (LLPS), Phase separation proteins (PSPs), Machine learning, Predictor

## Abstract

**Background:**

The liquid–liquid phase separation (LLPS) of biomolecules in cell underpins the formation of membraneless organelles, which are the condensates of protein, nucleic acid, or both, and play critical roles in cellular function. Dysregulation of LLPS is implicated in a number of diseases. Although the LLPS of biomolecules has been investigated intensively in recent years, the knowledge of the prevalence and distribution of phase separation proteins (PSPs) is still lag behind. Development of computational methods to predict PSPs is therefore of great importance for comprehensive understanding of the biological function of LLPS.

**Results:**

Based on the PSPs collected in LLPSDB, we developed a sequence-based prediction tool for LLPS proteins (PSPredictor), which is an attempt at general purpose of PSP prediction that does not depend on specific protein types. Our method combines the componential and sequential information during the protein embedding stage, and, adopts the machine learning algorithm for final predicting. The proposed method achieves a tenfold cross-validation accuracy of 94.71%, and outperforms previously reported PSPs prediction tools. For further applications, we built a user-friendly PSPredictor web server (http://www.pkumdl.cn/PSPredictor), which is accessible for prediction of potential PSPs.

**Conclusions:**

PSPredictor could identifie novel scaffold proteins for stress granules and predict PSPs candidates in the human genome for further study. For further applications, we built a user-friendly PSPredictor web server (http://www.pkumdl.cn/PSPredictor), which provides valuable information for potential PSPs recognition.

**Supplementary Information:**

The online version contains supplementary material available at 10.1186/s12859-022-04599-w.

## Background

The compartmentalization of molecules in the cytoplasm is critical for efficient and precise biochemical reactions in eukaryotic cells [42]. Studies on cellular compartments have traditionally been focused on membrane-bound organelles such as endoplasmic reticulum. However, membrane-less organelles, also called biomolecular condensates, have recently been recognized to compartmentalize cellular space through liquid–liquid phase separation (LLPS) [4, 15]. More and more studies suggest that many cellular metabolic processes are regulated by LLPS, so are some intractable diseases [30] such as ALS (amyotrophic lateral sclerosis) and AD (Alzheimer disease) [3]. Notably, several proteins are observed to form liquid-like membrane-less assemblies both in vitro and in vivo [7, 11, 15, 20, 35]. Studies also indicate that the LLPS of proteins and the formation of biomolecular condensates may be regulated by RNA [45].

LLPS in biology is deemed to be fundamentally driven by multivalent interactions between molecules, which can occur in proteins between multiple folded domains or are mediated by intrinsically disordered regions (IDRs). Generally, phase separation-related proteins can be categorized as scaffolds that drive LLPS or as clients that integrate into the condensates formed by scaffolds [10]. Although tremendous progress has been made in understanding protein LLPS, the knowledge of prevalence and distribution of phase separation proteins (PSPs), or specifically “scaffolds”, is still lacking. Development of computational methods to predict PSPs is therefore of great importance for deeper understanding the biological function of LLPS.

A recent review summarized a range of first-generation PSP prediction tools [39]. Each of these tools is based on specific protein features that are deemed to be driving forces behind LLPS. Specifically, PScore is based on the expected number of long-range, planar sp^2^ pi–pi contacts [38], the DDX4-like method is based on similarities in sequence composition and residue spacing to DDX4 [24], PLAAC is based on prion-like domains [2], LARKS is based on low-complexity aromatic-rich kinked segments [14], R + Y is based on the proportion of arginine and tyrosine, as well as features of FET family proteins [40], and CatGranule is based on the composition of amino acids that is responsible for granule formation [5]. Recently, FUS-LIKE PSPs were predicted using a hidden Markov model (HMM) that considered prion-like domains, disordered regions, arginine rich domains, RNA recognition motifs (RRM), and other features [25]. This tool, PSPer, has successfully predicted 22 experimentally studied FUS-LIKE proteins [40]. However, all these methods were based on small samples and specific features, limiting the scopes of their applications. Thus, large data-based prediction tools with more general application scopes are urgently needed.

An extremely powerful method for predicting protein function is machine learning. Prediction models can be trained by integrating aspects of protein features, including physical or chemical properties of residues or sequence context, as descriptors or vectors. Yet, development of PSP prediction tools using machine learning has been hampered by a lack of accumulated experimentally studied PSPs data. The publication of new PSP databases [21, 43, 44] is laying the groundwork for the creation of more general PSP prediction tools. A particularly promising example is the LLPS database (LLPSDB) [17], which is curated from published experiment results. Each entry in the database includes information about whether the protein (alone, with DNA/RNA, or with other proteins) undergoes phase separation under a specific in vitro experiment condition.

In this study, we developed a sequence-based machine learning PSP prediction tool (PSPredictor), based on data from LLPSDB. This new tool uses sequence information to make direct and more general predictions about proteins undergoing LLPS. Our model achieved a tenfold cross-validation training accuracy of 94.71% and a prediction accuracy of 92.50% on an external test set. PSPredictor also performed much better than the reported first-generation PSP prediction tools in identifying new PSPs.

## Implementation

### Dataset construction for PSP prediction

The LLPSDB is a valuable resource for constructing data-driven machine learning models [17], because it records the detailed information about proteins undergoing LLPS in specific experimental conditions. For model training, we selected the sequences from the LLPSDB as positive dataset (see Transparent methods). We obtained a total of 353 protein sequences and selected 293 protein sequences from the initial version of LLPSDB as a positive training dataset P1 for primary model construction. We used the remaining 60 protein sequences from final release version of LLPSDB as an external test dataset (T1+). Then we used all 353 protein sequences (dataset P) as the positive training dataset to construct the final model for the PSPredictor tool.

As LLPS is deemed to be driven by multivalent interactions between multiple folded domains or disordered domains, we used the PDB databank to select single-domain proteins with full-length and solved three-dimensional (3D)-structures. A total of 5258 protein sequences were screened as the negative training dataset (N1). Due to the imbalance issue of the dataset, we conducted undersampling to selected samples from N1 for model training by random sampling to ensure the scientificalness and rationality of the research. The undersampling is operated by different ratio to learn the best composition to construct the predictor in this scenario.

## Methods

All methods could be found in the accompanying Transparent Methods in Additional file 1.

## Results

### Development of the PSP prediction tool—PSPredictor

To train the primary model and identify which model performed best, we systematically combined three categories of variables. These categories included:

1. During the undersampling stage, ratios between positive and negative training samples are 1:1, 1:2 and 1:5;
2. Selected protein coding methods (evolutionary word2vec (w2v), Li’s method (LQL)) [18, 22];
3. Machine learning algorithms (K-Nearest Neighbor (KNN), Supported Vector Machine (SVM), Random Forest (RF), Logistic Regression (LR), Decision Tree (DT), Gradient Boosting Decision Tree (GBDT), Naive Bayes (NB)).

Combining these variables resulted in a total of 42 (3 × 2 × 7) models. Based on the evaluation of the statistical indexes of Accuracy, F1, Precision, Sensitivity, Specificity and MCC, the best model (model 1) was selected (All models’ training results can be found in Additional file 2: Table S1) and the significant differences between model 1 and others have been assessed by paired t-test (*p* value < 0.05). The model 1 is w2v coded, trained by GBDT, and the ratio between positive and negative samples is 1:1. It achieved a tenfold cross-validation training accuracy of 94.71% ± 2.54% (the training statistical index values are shown in Table 1). As for different model with same sample ratio and feature descriptor, the best algorithm to predict the LLP is GBDT, and, the w2v is proved to be better than LQL with same sample ratio and machine learning algorithm.

**Table 1:** The evaluation of the best model (model 1) for PSPs prediction

Accuracy^a^	F1^a^	Precision^a^	Sensitivity^a^	Specificity^a^	MCC^a^
0.95 ± 0.03	0.92 ± 0.01	0.95 ± 0.03	0.94 ± 0.04	0.96 ± 0.05	0.90 ± 0.05

^a^Data are represented as mean ± SD

Because the negative samples were selected from dataset N1 by random sampling, we independently repeated the training process for three times, all the training results were similar (Additional file 2: Table S2). Additional details about the construction of training datasets, protein coding methods, machine learning algorithms, and definition of statistical indexes can be found in the Transparent methods.

We selected the first trained best model (model 1) as the primary model to conduct predicting on the external dataset (dataset T1+). 95% proteins in T1+ were identified as PSPs by model 1. For 13 protein sequences in T1+ that share less than 30% sequence similarity with those in P1, 11 of them were predicted as PSPs by model 1. We also used dataset N1, excluding the sequences in the negative training dataset, as an external negative test set to avoid the risk of over-fitting, it is found that the prediction accuracy was 92.50% and the Brier score loss is found as 0.0917.

**Fig. 1:**
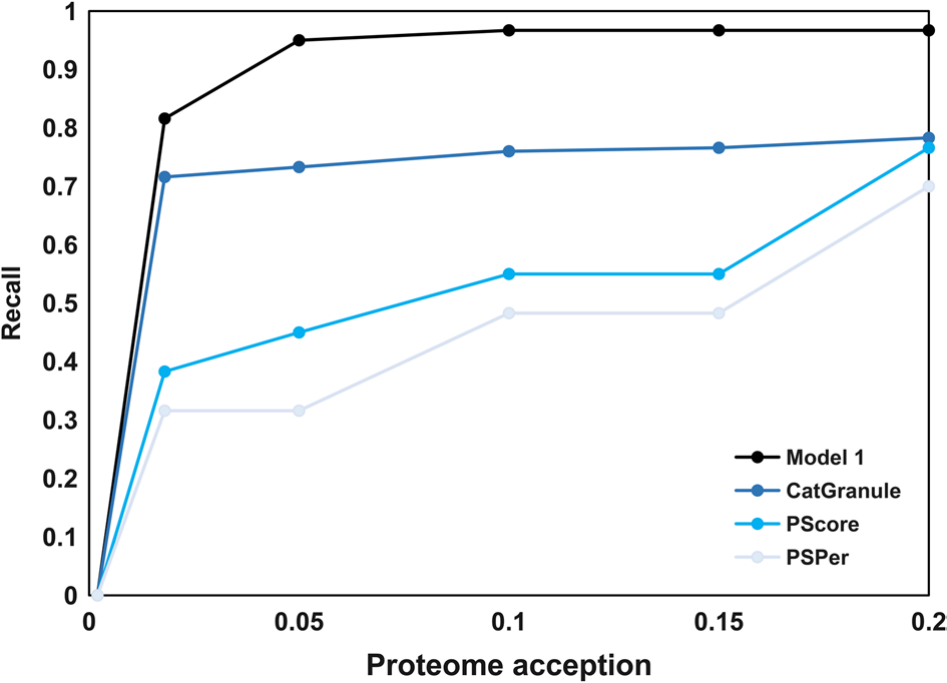
Relationship between percent recall and total percentage of human proteins accepted at given thresholds, for Model 0 and three best first generation prediction tools

We compared our testing results with two published first-generation PSP prediction tools, PScore [38] and CatGranule [5], which performed best among the 7 first-generation methods on a benchmark dataset [39], as well as PSPer [25] for the prediction of dataset T1+. Figure 1 shows the relationships between percent recall and total percentage of whole proteins accepted at given thresholds for PScore, CatGranule, PSPer, and our model 1. Obviously, our model 1 is superior to other models in dataset T1+ prediction.

We combined dataset P1 and T1+ together as a full positive training dataset (P), and used the same negative dataset and parameters of model 1 to train a model, that is, PSPredictor as our final PSP prediction model.

### Analysis of scaffolds, regulators, clients in DrLLPS, RNA granule and P-body forming proteins

**Fig. 2:**
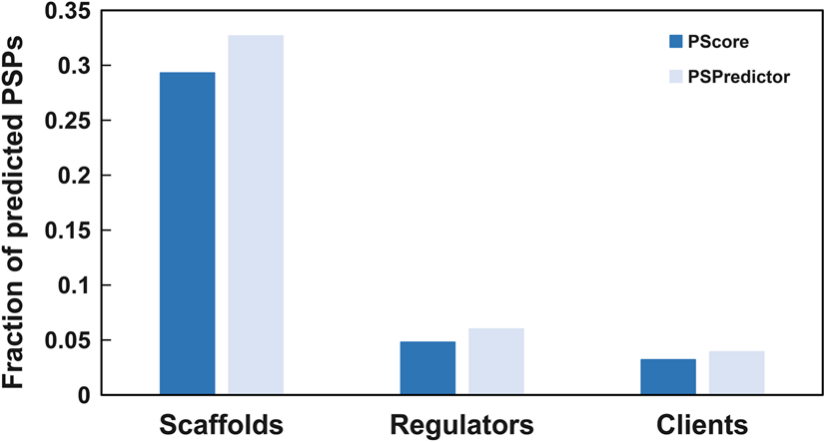
Fraction of proteins in each category (scaffold, regulator or client) predicted as PSPs by PSPredictor or PScore

Proteins involved in LLPS can be categorized as scaffolds and clients. Scaffolds are defined as the drivers of LLPS, whereas clients have been discovered to coalescence with scaffolds in experimental conditions. A recently published database, DrLLPS [23] added another category of LLPS-related protein, called regulators. Regulators were defined as regulating LLPS behaviors of scaffolds by various mechanisms, such as post-translational modification. However, these categories of proteins sometimes are overlapped, meaning that individual protein may act as a scaffold, regulator or client, depending on the context [1]. We used PSPredictor to estimate real PSPs, defined here as proteins that can undergo LLPS independently or with DNA/RNA. At a high threshold (1.8%), PSPredictor predicted that 32.7% were PSPs, whereas only 6% regulators and 3.92% clients are predicted as PSPs. Also, the proportions of PSPs predicted by PSPredictor are higher than those predicted by PScore (Fig. 2).

**Fig. 3:**
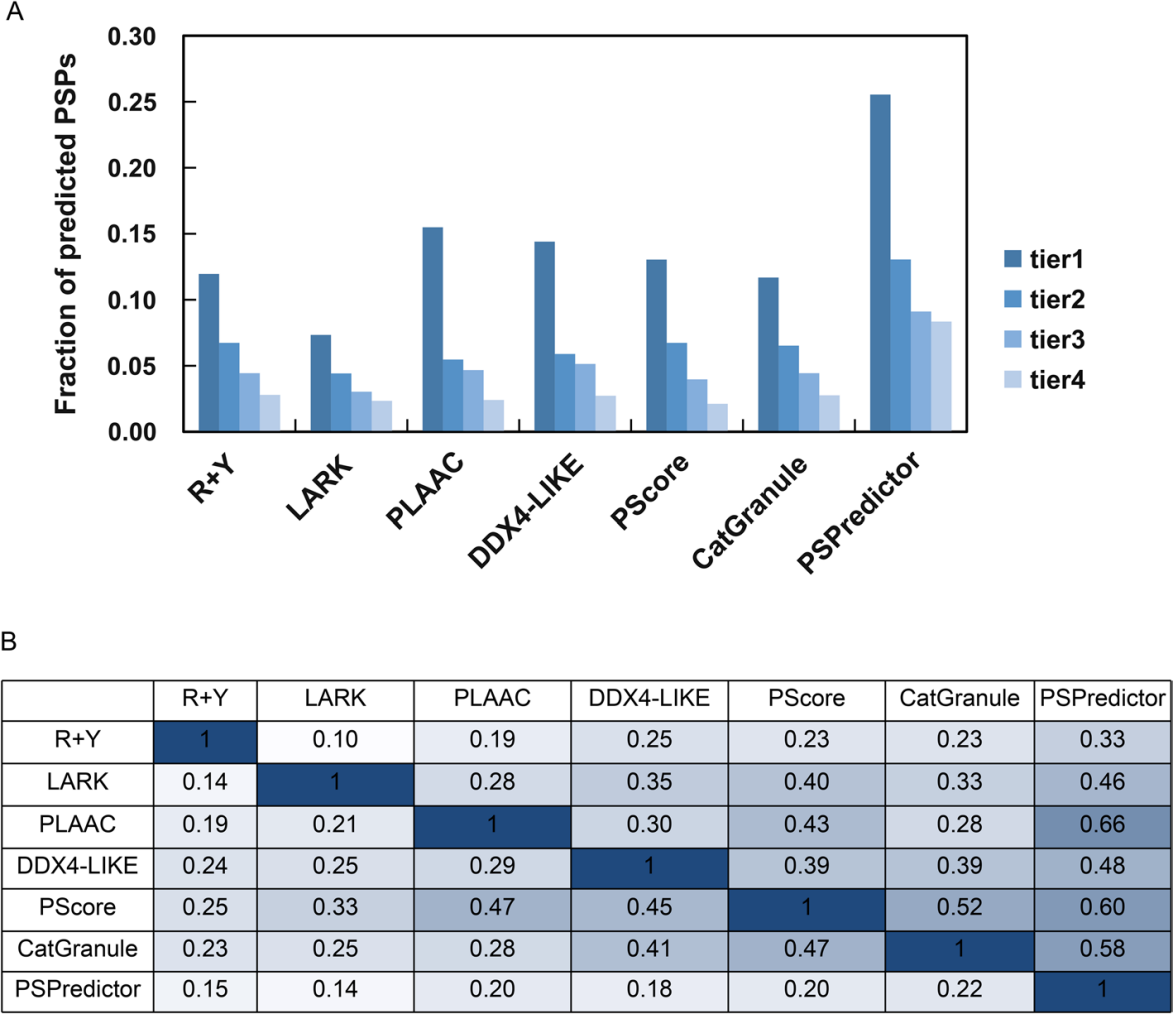
**A** Fraction of proteins in each tier group predicted as PSPs by first generation prediction tools and PSPredictor. **B** The number of predicted PSPs that overlapped between two prediction tools

It is also unknown whether some of the proteins, which are the core components in stress granules or P-body condensates can undergo LLPS independently. Recently, Youn et al. published a comprehensive database of proteins related to the formation of stress granules and P-body condensates. Each protein (4385 sequences total) was assigned to a tier of 1–4, according to the degree of confidence for whether the protein localized in stress granules or P-bodies [44]. We analyzed these proteins using PSPredictor and compared results for each tier with results obtained using reported PSP prediction tools (Fig. 3A). We also calculated the number of predicted PSPs that overlapped between any two tools (Fig. 3B). For all the prediction tools, the proportion of predicted PSPs ranked as tier 1 > tier 2 > tier 3 > tier 4, which is consistent with the degree of confidence assigned by Youn et al. PSPredictor predicted more PSPs than other tools and had the most overlapped number of PSPs with those predicted by other tools. For all the proteins in the database reported by Youn et al., PSPredictor indicated that 10.37% of proteins in stress granules or P-bodies may spontaneously undergo LLPS, compared to other tools that gave an estimation of ~ 3–4%.

These results emphasize that only a small proportion of proteins spontaneously forming condensates [1, 25] as scaffolds, and a large proportion of proteins in the RNA granules might participate in the phase separated condensates as clients.

### Scanning the human genome for potential PSP

Human proteins in the top 1.8% (high confidential threshold) of the human proteome predicted by PSPredictor are regarded as consolidated PSPs. We performed gene ontology (GO) term enrichment analysis on these predicted PSPs. Terms with EASE score < 0.1 are shown in Additional file 1: Table S3 (see Additional Information). Most of the GO terms that were identified by first-generation PSP prediction tools [39], are also enriched in our predicted consolidated PSPs. These terms included “cytoplasmic stress granule”, “intracellular ribonucleoprotein complex”, etc. Comparing with the first-generation tools, nucleus associated PSPs are more likely to be identified by PSPredictor. The human PSPs predicted by PSPredictor are available at https://github.com/pkumdl/PSPredictor.

When we clustered GO terms with a similar biological context, we observed 7 clusters with high enrichment scores (Additional file 1: Table S4) such as proteins in the *mRNA processing, mRNA splicing, mRNA processing* cluster, which is agreeing with the finding of recent research that the concerning features of mRNA regulate and influence the LLPS characteristics [29]. Corresponding to the clustering results, it has been demonstrated that multiple zinc-binding sites on specific protein are involved in the LLPS-promoting effect [33]. The DNA-Binding and RNA-binding proteins related to liquid–liquid phase separation has been widely discussed as well [13, 32], suggesting that PSPredictor results can provide the clue of functional studies for newly predicted PSPs.

### PSPredictor webserver

We constructed a web server for online PSPredictor computation (http://www.pkumdl.cn/PSPredictor). Through this portal, users can upload their protein sequences and predict if they are PSPs or not. For the query sequences, the NCBI blast tool [8] is embedded to search for similar sequences collected in LLPSDB, which can further link to LLPSDB for more information about the phase behavior and biological function of the related proteins. Additional file 1: Figure S1 shows the main page and an example of the computational output of the web server.

## Discussion

### GBDT is an efficient machine learning algorithm for PSP prediction

We tested seven machine learning algorithms: SVM, KNN, RF, LR, DT, GBDT and NB (see Transparent methods in Additional file 1), on their ability to predict PSPs when combined with two types of encoding methods. Most of our best models were obtained by training with GBDT, a powerful and widely used supervised machine learning algorithms. GBDT integrates gradient boosting and decision trees and is capable of both linear and nonlinear data classification, regression, and prediction. GBDT can generalize and combine weak learners into a single, strong learner and has produced good results in biological data mining compared to other machine learning algorithms [16, 19, 27, 34, 41]. Our research is another successful application of GBDT in biology. We did not test other deep learning algorithms due to the limited size of the current dataset. This could be tested in the future with increasing data size.

### W2v captures PSP sequence features and performs well in PSP prediction

W2v is a natural language processing technique by which words are embedded in vectors through the training of contexts. They could also embed residues, protein and chemicals into vectors as inputs for model training without requiring artificial feature design or expert knowledge. It had been successfully used to predict HLA binding proteins, antimicrobial peptides and drug targets [12, 31, 34, 36].

**Fig. 4:**
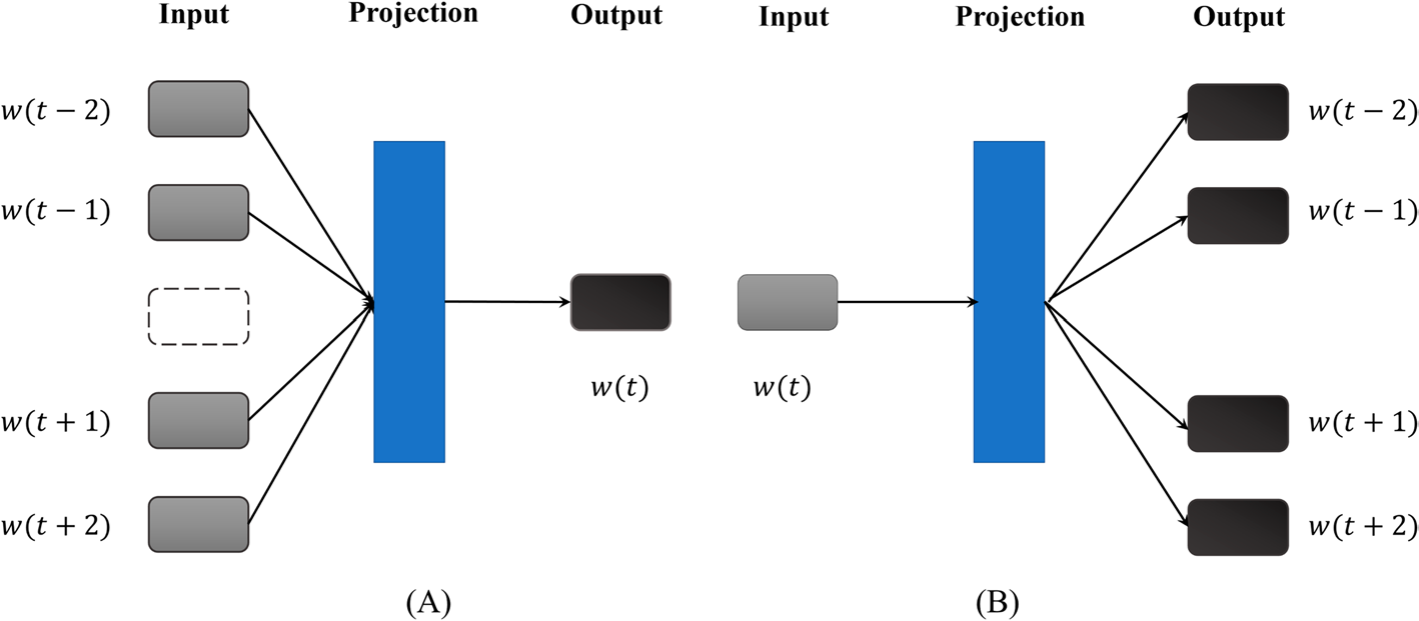
The model architectures of CBOW (**A**) and Skip-gram (**B**)

W2v is developed on basic of Neural Network Language Model [6]. In order to improve the computing speed of traditional method, the nonlinear hidden layer in the feedforward feedback neural network is removed, and the middle embedding layer is directly connected to the output layer. W2v includes two learning algorithms, namely continuous bag-of-word (CBOW) and skip-gram algorithms. Figure 4 shows the model architectures of CBOW and Skip-gram. CBOW uses a Huffman tree to maximize the conditional log-likelihood, whereas the skip-gram model minimizes the log-likelihood of sampled negative instances. In this work, Skip-gram model with window size 8, and hierarchical softmax were recruited. We downloaded the entire protein sequences from swiss-prot, and broke the original sequences into 3 residue-length windows overlapped kmers. The dimension was set to 200. We used w2v program in genism python NLP package [28] (https://radimrehurek.com/gensim/) to train and compute the embedding vectors.

In order to further ensure that w2v could capture the information of protein sequence pattern, not the amino acid composition, we add the position encoding operation to use the order the sequences. In this paper, we adopt Vaswani’ s method [37] to use sine and cosine functions of different frequencies as follows:

$$\begin{aligned} {PE}_{(pos,2i)}&=\mathit{sin}\left(pos/{10000}^{2i/{d}_{model}}\right)\\ {PE}_{(pos,2i+1)}&=\mathit{cos}\left(pos/{10000}^{2i/{d}_{model}}\right) \end{aligned}$$

where $pos$ is the position and $i$ is the dimension, $PE$ is relative positional encoding result. Each dimension of the positional encoding corresponds to a sinusoid, and, the wavelengths form a geometric progression from $2\pi$ to $10000\cdot 2\pi$. For any offset k, ${PE}_{pos+k}$ could be represented as a linear function of ${PE}_{pos}$.

By adopting this method, the componential and sequential information the protein could be including in the embedding vector, simultaneously.

To further validate the effectiveness of our method, we transformed the protein sequences into samples with variables of 20 kinds of amino acid content and sequence length. With such a total of 21-dimensional input variables, we constructed model Com-Len and conducted the PSPs predicting, and the corresponding results are shown in Table 2. Obviously, the accuracy of Com-Len is 87.44% which is far less than the model 1 (significant different is assessed by paired t-test with *p* < 0.0001). Furthermore, we conducted random shuffling on each protein sequence in dataset T1+ for 100 times, respectively (the Shuffled Dataset including 60 × 100 = 6000 generated sequences), and then predicted the liquid–liquid phase behavior for each of them through model 1 (with ratio of negative and positive samples is 1, and, GBDT predictor). 80.30% ± 4.53% of the shuffled sequences are predicted to be PSPs, that means, the model works to capture the composition of the PSPs, which is close to the accuracy of Com-Len predictor (as shown in Table 2). It also illustrates besides the composition of amino acids in protein sequence, our model could also capture the sequence pattern as well.

**Table 2:** The evaluation of the Com-Len model for PSPs prediction

Accuracy^a^	F1^a^	Precision^a^	Sensitivity^a^	Specificity^a^	MCC^a^
0.87 ± 0.04	0.87 ± 0.04	0.92 ± 0.04	0.81 ± 0.06	0.93 ± 0.04	0.76 ± 0.07

^a^Data are represented as mean ± SD

**Fig. 5:**
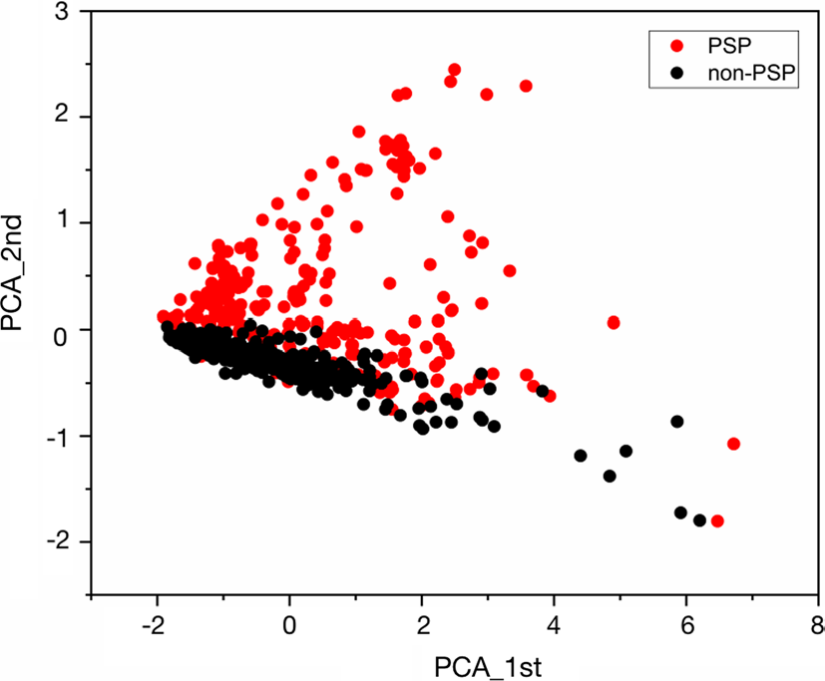
2D vector projection of PSPs and non-PSPs by PCA

In order to visualize the embedding space of PSPs, we reduced the dimensionality of the protein-space of the training datasets using principle component analysis (PCA). The first two dimensions explained 70% of the varieties (1st: 59%, 2nd: 11%). We then generated the two-dimensional (2D) scatter plot for PSPs and non-PSPs (as shown in Fig. 4, the distribution patterns of the other two repeat trainings with different negative samples are shown in Additional file 1: Fig. S2). It could be seen that PSPs and non-PSPs were separated well in 2D w2v space after PCA, indicating that w2v could capture sequence features of PSPs (Fig. 5).

### Datasets used for training PSPredictor

For our positive training dataset, we used the PSPs in LLPSDB that can form LLPS independently or with DNA/RNA, whereas the negative training dataset contained single-domain proteins with full-length, solved 3D structures. To rule out the possibility that our model is not a kind of IDRs prediction model, we compared the prediction scores for proteins in the dataset T1+ and IDPs from Disprot. We found significant differences (*p* < 0.01) between the scores of two protein groups, and only 31% of the proteins in the IDP dataset were predicted to be PSPs (see Transparent methods). These results indicate that PSPredictor is not an IDRs prediction model. As the positive dataset includes both multi-domain proteins such as FUS [26], TDP-43 [9], and short cleaved single domain proteins (such as FUS RGG domain) or designed repeated peptides (such as ( R)_20_), all these incorporated protein features imply PSPredictor would be more general for the prediction of PSPs than other reported tools which are specific feature-based.

### Limitations of the study and perspectives for future PSP prediction

We have shown that the data in the LLPSDB make it possible to develop a universal PSP prediction model that is not restricted to a few specific protein domains. Previously, the limited availability of experimental data dictated that most first-generation PSP prediction tools were dependent on specific features. As PSP data accumulates, we expect that predictive tool like PSPredictor will cover more PSP space with highly accurate predictions. Other data-demanding algorithms, like various deep learning methods, could be employed in appropriate situations in the future. Currently, PSPredictor and first-generation prediction tools could be used to predict driver proteins, whether PSP client proteins need specialized prediction tools or generalized tools can be developed need further investigation.

Generally speaking, all proteins can undergo LLPS in correct conditions. In our positive dataset, we only included PSPs that form LLPS in near physiological conditions without considering their environmental differences. With more data available, experimental conditions may be integrated in future training processes, so that PSP can be predicted for various temperature, salt, pH, and crowding conditions. Another challenge is to predict PSP mutants that inhibit or prevent LLPS. Due to the high level of sequence similarity, it is difficult for sequence that based prediction tools to differentiate them. Besides, current PSPredictor is not considering PTMs, therefore it is not suitable for identifying the regulation of PTMs now. More data and sophisticated model may be required for all the above kinds of prediction.

## Conclusions

In this work, adopt the evolutionary word2vec and Machine Learning algorithm for PSPs predicting. By cross-validation and comparison experiment, it demonstrated that the proposed PSPredictor could identify the componential and sequential information at the same time. PSPredictor identifies novel scaffold proteins for stress granules and predicts PSPs candidates in the human genome for further study. And, the accessible PSPredictor web server provides valuable information for potential PSPs recognition.

### Availability and requirements

- Project name: Prediction of liquid–liquid phase separating proteins using machine learning (PSPredictor)
- Project home page: http://www.pkumdl.cn/PSPredictor
- Operating system(s): Win, Mac and Linux
- Programming language: Python, PHP
- Other requirements: Apache 2.2.15
- License: Academic Free License
- Any restrictions to use by non-academics: license needed.

## Supplementary Information

**Additional file 1: Fig. S1.** Snapshot of main page of PSPredictor web server; **Fig. S2.** PCA 2D projection of PSPs and non-PSPs with three different sets of negative samples; **Table S3.** Enrichment GO terms of human PSPs predicted by PSPredictor; **Table S4.** GO term clusters of PSPs with similar meaning in biology.

**Additional file 2:**  **Table S1.** All models’ training results; **Table S2.** The training results of three repeats of models with (1) w2v coded, (2) the ratio of positive samples and negative samples is 1:1, (3) sequence number is 586, and, (4) GBDT trained.

## Data Availability

The link of the adopted dataset LLPSDB is http://bio-comp.org.cn/llpsdb/, and, the human PSPs predicted by PSPredictor are available at https://github.com/pkumdl/PSPredictor.

## Abbreviations

LLPS: The liquid–liquid phase separation
PSPs: Phase separation proteins
LLPSDB: A database of proteins undergoing liquid–liquid phase separation in vitro (please see details in http://bio-comp.org.cn/llpsdb/)
PSPredictor: The proposed sequence-based prediction tool for LLPS proteins
ALS: Amyotrophic lateral sclerosis
AD: Alzheimer disease
RNA: Ribonucleic acid
DNA: Deoxyribonucleic acid
IDRs: Intrinsically disordered regions
PScore, PLAAC, LARKS, R + Y, FUS-LIKE PSPs, CatGranule, PSPer: Existing PSP prediction tools
HMM: Hidden Markov model
RRM: RNA recognition motifs
P: The full positive training dataset proposed in this paper
P1: The positive training dataset proposed in this paper
T1+: The external test dataset proposed in this paper
N1: The negative training dataset proposed in this paper
w2v: The proposed embedding method in this study
LQL: A comparing method proposed by Li and Lai in the previous research [18]
KNN: K-nearest neighbor
SVM: Supported vector machine
RF: Random forest
LR: Logistic regression
DT: Decision tree
GBDT: Gradient Boosting Decision Tree
NB: Naive Bayes
NLP: Natural language processing
PCA: Principle component analysis
FUS: Fused in sarcoma
TDP-43: TAR DNA-binding protein 43
PTMs: Parathymosin
