# Supplementary material for: Prediction of liquid–liquid phase separating proteins using machine learning
Source: BMC Bioinformatics. 2022 Feb 15;23:72. doi: 10.1186/s12859-022-04599-w (PMC8845408; doi:10.1186/s12859-022-04599-w)
Supplement: Supplementary file 1 — Additional file 1: Fig. S1. Snapshot of main page of PSPredictor web server; Fig. S2. PCA 2D projection of PSPs and non-PSPs with three different sets of negative samples; Table S3. Enrichment GO terms of human PSPs predicted by PSPredictor; Table S4. GO term clusters of PSPs with similar meaning in biology. [file 12859_2022_4599_MOESM1_ESM.docx]

# Supplemental Information

**Supplemental Figures and Figure Legends**


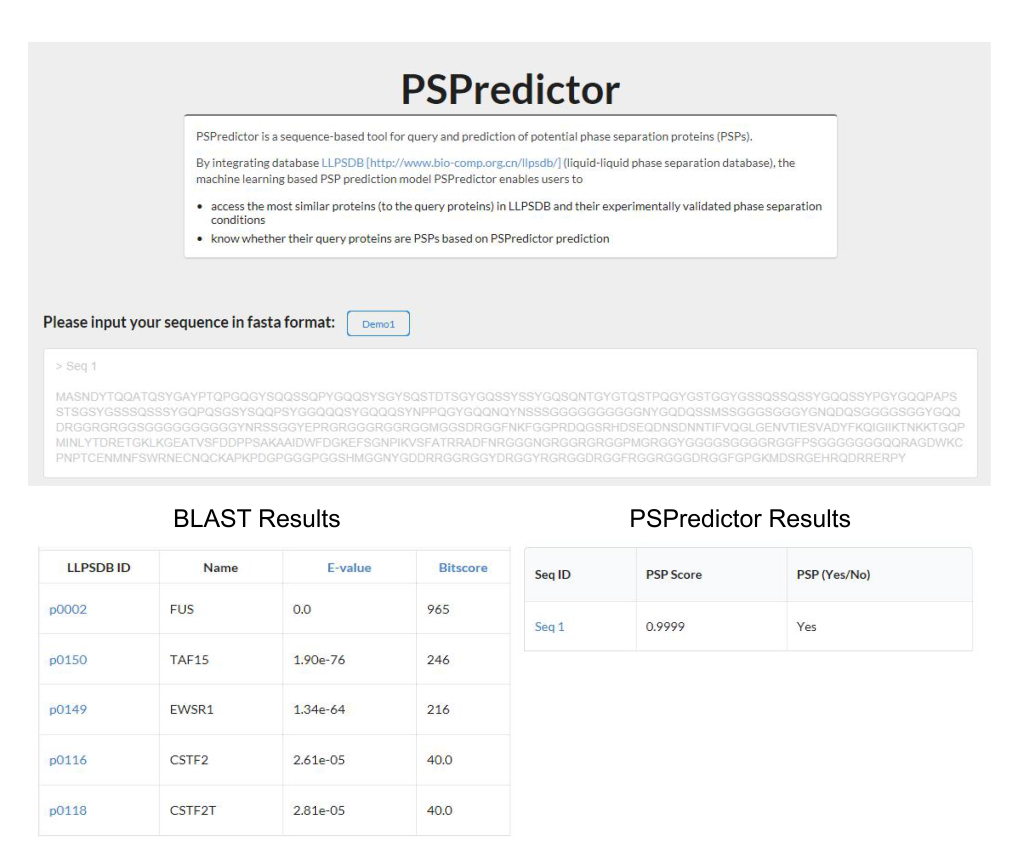


**Fig. S 1.** Snapshot of main page of PSPredictor web server.


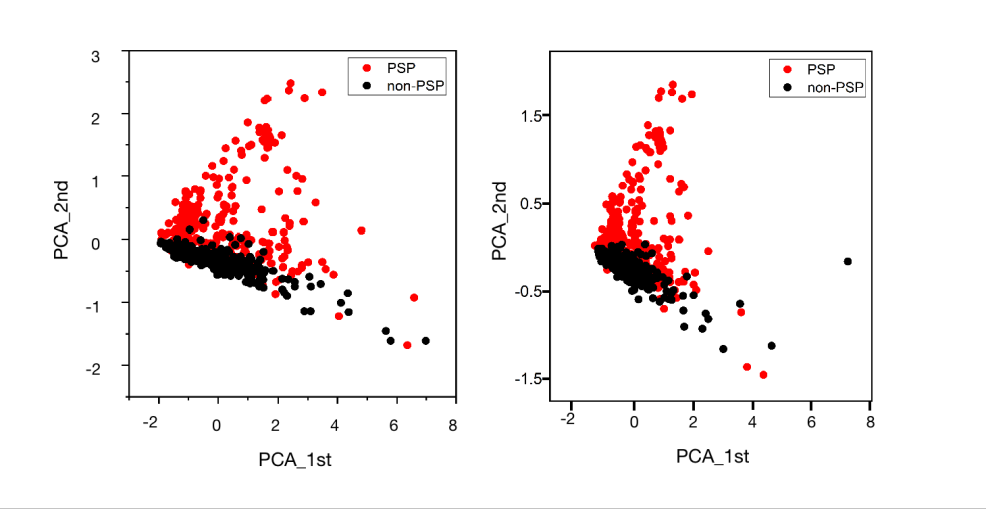


**Fig. S 2** PCA 2D projection of PSPs and non-PSPs with three different sets of negative samples

**Supplemental Tables and Table Legends**

**Table S 3.** Enrichment GO terms of human PSPs predicted by PSPredictor

| **GO terms** | **Percent (%)**a | **P-Value**b |
| --- | --- | --- |
| nucleus | 53.2 | 6.40E-57 |
| nucleoplasm | 34.9 | 2.90E-50 |
| nuclear speck | 5.4 | 1.50E-17 |
| cytoplasm | 38.9 | 4.10E-16 |
| intracellular ribonucleoprotein complex | 3.6 | 1.20E-11 |
| nucleolus | 9.1 | 5.80E-09 |
| cytoplasmic stress granule | 1.7 | 2.70E-08 |
| transcription factor complex | 3.3 | 3.80E-07 |
| nuclear chromatin | 3 | 5.20E-06 |
| heterochromatin | 1 | 6.70E-05 |
| actin cytoskeleton | 2.8 | 2.90E-04 |
| viral nucleocapsid | 1 | 3.70E-04 |
| catalytic step 2 spliceosome | 1.7 | 4.00E-04 |
| intracellular membrane-bounded organelle | 5.1 | 4.20E-04 |
| BAF-type complex | 0.6 | 8.20E-04 |
| nuclear pore central transport channel | 0.7 | 8.70E-04 |
| RNA polymerase II transcription factor complex | 1 | 9.50E-04 |
| chromosome | 1.7 | 1.20E-03 |
| ruffle | 1.5 | 1.30E-03 |
| lamellipodium | 2.1 | 1.70E-03 |
| cytoplasmic mRNA processing body | 1.4 | 1.70E-03 |
| spliceosomal complex | 1.5 | 1.80E-03 |
| intermediate filament | 1.7 | 2.20E-03 |
| PcG protein complex | 0.8 | 2.30E-03 |
| keratin filament | 1.5 | 2.90E-03 |
| actin cortical patch | 0.4 | 3.70E-03 |
| exon-exon junction complex | 0.7 | 6.90E-03 |
| dendritic growth cone | 0.4 | 7.20E-03 |
| chromocenter | 0.6 | 7.70E-03 |
| PML body | 1.4 | 8.00E-03 |
| Cajal body | 1 | 9.00E-03 |
| PRC1 complex | 0.6 | 9.70E-03 |
| polysome | 0.8 | 1.10E-02 |
| cell-cell adherens junction | 2.9 | 1.10E-02 |
| mRNA cleavage factor complex | 0.4 | 1.20E-02 |
| focal adhesion | 3.3 | 1.30E-02 |
| transcriptional repressor complex | 1 | 1.30E-02 |
| cell projection | 1.1 | 1.70E-02 |
| messenger ribonucleoprotein complex | 0.4 | 1.70E-02 |
| postsynaptic density | 1.8 | 3.10E-02 |
| microtubule associated complex | 0.7 | 3.10E-02 |
| nuclear membrane | 2.1 | 3.40E-02 |
| MLL3/4 complex | 0.4 | 3.80E-02 |
| stress fiber | 0.8 | 4.20E-02 |
| neuron projection | 2.1 | 4.30E-02 |
| transcription elongation factor complex | 0.6 | 4.60E-02 |
| histone methyltransferase complex | 0.6 | 4.60E-02 |
| bicellular tight junction | 1.2 | 4.80E-02 |
| growth cone | 1.2 | 5.40E-02 |
| nuclear matrix | 1.1 | 5.70E-02 |
| protein-DNA complex | 0.6 | 5.70E-02 |
| cytoplasmic ribonucleoprotein granule | 0.6 | 5.70E-02 |
| synapse | 1.7 | 5.80E-02 |
| nuclear pore nuclear basket | 0.4 | 6.50E-02 |
| nuclear euchromatin | 0.6 | 6.90E-02 |
| cornified envelope | 0.7 | 7.90E-02 |
| actin filament | 0.8 | 8.00E-02 |
| nBAF complex | 0.4 | 8.60E-02 |

a. Percent is the ratio of the number of proteins that were annotated with the GO terms and the total number of proteins

b. p-value is a modified Fisher Exact P-Value, for gene-enrichment analysis. It ranges from 0 to 1. Fisher Exact P-Value = 0 represents perfect enrichment. Usually P-Value is equal or smaller than 0.05 to be considered strongly enriched in the annotation categories.

**Table S 4**. GO term clusters of PSPs with similar meaning in biology

| Annotation Cluster 1 | Enrichment Score: 37.5 | Term explains | P_Value |
| --- | --- | --- | --- |
|  | UP_KEYWORDS | Transcription | 1.30E-45 |
|  | UP_KEYWORDS | Transcription regulation | 2.30E-45 |
|  | GOTERM_BP_DIRECT | transcription, DNA-templated | 2.60E-31 |
|  | UP_KEYWORDS | DNA-binding | 1.30E-29 |
| Annotation Cluster 2 | Enrichment Score: 16.53 |  | P_Value |
|  | UP_KEYWORDS | RNA-binding | 5.20E-31 |
|  | INTERPRO | RNA recognition motif domain | 4.60E-24 |
|  | INTERPRO | Nucleotide-binding, alpha-beta plait | 1.70E-23 |
|  | SMART | RRM | 1.70E-19 |
|  | GOTERM_MF_DIRECT | nucleotide binding | 6.00E-17 |
|  | UP_SEQ_FEATURE | domain:RRM | 2.80E-14 |
|  | GOTERM_MF_DIRECT | nucleic acid binding | 5.30E-09 |
|  | UP_SEQ_FEATURE | domain:RRM 1 | 1.60E-08 |
|  | UP_SEQ_FEATURE | domain:RRM 2 | 1.60E-08 |
| Annotation Cluster 3 | Enrichment Score: 13.01 |  | P_Value |
|  | UP_KEYWORDS | mRNA processing | 3.50E-16 |
|  | UP_KEYWORDS | mRNA splicing | 5.70E-16 |
|  | GOTERM_BP_DIRECT | mRNA processing | 5.70E-14 |
|  | GOTERM_BP_DIRECT | RNA splicing | 1.60E-12 |
|  | GOTERM_BP_DIRECT | mRNA splicing, via spliceosome | 5.00E-10 |
| Annotation Cluster 4 | Enrichment Score: 11.23 |  | P_Value |
|  | UP_KEYWORDS | Zinc-finger | 3.20E-19 |
|  | UP_KEYWORDS | Zinc | 2.20E-14 |
|  | GOTERM_MF_DIRECT | zinc ion binding | 1.90E-09 |
|  | UP_KEYWORDS | Metal-binding | 8.90E-05 |
| Annotation Cluster 5 | Enrichment Score: 5.9 |  | P_Value |
|  | GOTERM_BP_DIRECT | mRNA splicing, via spliceosome | 5.00E-10 |
|  | KEGG_PATHWAY | Spliceosome | 2.30E-08 |
|  | GOTERM_CC_DIRECT | catalytic step 2 spliceosome | 4.00E-04 |
|  | UP_KEYWORDS | Spliceosome | 5.20E-04 |
| Annotation Cluster 6 | Enrichment Score: 5.37 |  | P_Value |
|  | INTERPRO | High mobility group (HMG) box domain | 6.00E-07 |
|  | SMART | HMG | 6.50E-06 |
|  | UP_SEQ_FEATURE | DNA-binding region:HMG box | 1.90E-05 |
| Annotation Cluster 7 | Enrichment Score: 5.20 |  | P_Value |
|  | GOTERM_BP_DIRECT | RNA export from nucleus | 5.80E-08 |
|  | GOTERM_BP_DIRECT | mRNA export from nucleus | 5.70E-07 |
|  | GOTERM_BP_DIRECT | mRNA 3'-end processing | 1.30E-06 |
|  | GOTERM_BP_DIRECT | termination of RNA polymerase II transcription | 1.70E-05 |
|  | KEGG_PATHWAY | mRNA surveillance pathway | 1.30E-02 |

**Transparent Methods**

In this paper, we defined PSPs as proteins that can undergo LLPS independently or with DNA/RNA. For the positive training datasets, we selected those entries in which protein could undergo LLPS independently or with DNA/RNA. These proteins have ≥50 residues and all are IDR-contained. Due to that the post translation modifications (PTMs) of protein in LLPSDB are only marked in annotation but not in recorded sequences, those entries with PTMs are not selected. After removing the duplicate sequences (As some proteins can undergo LLPS both by themselves and with DNA/RNA), we obtained 353 positive samples (dataset P), which included 102 proteins with whole sequence disordered and 251 proteins with IDR-fold structure. Approximately 78.8% of the positive samples were observed to undergo LLPS under physiological condition (excluding those with temperature < 0 °C or >40 °C; or salt concentration < 50mM, or >200mM; or pH < 5 or >8). Based on the 353 protein sequences, we constructed training and external test datasets.

**Positive Dataset P:** The positive training dataset for training the final model. The total 353 protein sequences from LLPSDB selected as previously illustrated. **Positive Dataset P1:** To train the primary model, we selected 293 protein sequences from dataset P, based on the release version of LLPSDB (from the initial release version of LLPSDB updated in May 10th 2019).

**Positive Dataset T1+:** From dataset P2, a total of 60 protein sequences were selected as external test dataset for the primary model (from the final release version of LLPSDB updated in July 12^th^ 2019).

**Negative Dataset N1:** Protein sequences which were unlikely to undergo LLPS. For candidate negative dataset, we downloaded sequences from the PDB (Burley et al., 2018) and selected those with 3D-structures that encompassed the full-length sequences. There were 14778 of such protein sequences. We removed redundancy so that no two sequences in the dataset had a sequence similarity of ≥ 50%. A total of 8625 protein sequences were obtained. Then, we used Hmmer3 for domains prediction, and selected single domain protein sequences as Negative Dataset N1 (5258 sequences).

When N1 was used for training, we selected an equal, 2-fold, or 5-fold (relative to the number of positive samples) number of protein sequences from dataset N1 as negative training samples by approach of random sampling.

**Youn’s dataset:** This dataset contains 4385 stress granule or P-body related proteins. These proteins were assigned to 4 tiers based on the quality and sufficiency of evidences that proteins locating in stress granule or P-body. Tier 1 contained 368 proteins, tier 2 contained 475 proteins, tier 3 contained 428 proteins and tier 4 contained 3114 proteins (Youn et al., 2019).

**DrLLPS**: This database contains 150 scaffolds, 986 regulators and 8148 clients(Ning et al., 2019). They were collected from literatures.

Protein coding

We tested different protein sequence coding methods for predicting protein-protein interaction and protein drug target. Our work showed that the LQL method and w2v method capture important features and performed well in model training and prediction in the above tasks (Sun et al., 2018; Sun et al., 2017).

LQL method: In LQL method (Li and Lai, 2007), the composition of 20 amino acids formed the first 20 dimensions. Amino acids were then clustered into three types for each of six physicochemical properties (hydrophobicity, polarity, polarizability, solvent accessibility, and normalized van der Waals volume). We calculated “Composition,” “Transition,” and “Distribution” for each type and for each property. For example, amino acids were clustered into polar, neutral and hydrophobic for hydrophobicity property. For ‘Composition’, 3 dimensions were calculated: the percentage of polar, neutral and hydrophobic; For ‘Transition’, 3 dimensions were calculated: the percentage of polar transferred to neutral, neutral transferred to hydrophobicity and hydrophobicity transferred to polar; For ‘Distribution’, 5 dimensions were calculated for each of the 3 types of amino acids: the location percentage of the first, 25%, 50%, 75% of that type. A vector of total 146 dimensions was calculated for each protein.

Evolutionary word2vec (w2v): W2v (Mikolov et al., 2013) is the name of a series of models that are trained to produce word embedding vectors. Two models are popular: continuous bag-of-words (CBOW) and continuous skip-gram. CBOW predicts a current word from a window surrounding the context words, whereas the skip-gram model uses a current word to predict a surrounding window of context words. Hierarchical softmax and negative sampling are the two main training methods. In this work, Skip-gram model with window size 8, and hierarchical softmax were recruited. Meanwhile, to further use the order of the sequences, the computational results of the positional encoding are added into embedding vectors (Vaswani, et al., 2017) in this work. After obtaining the embedding vectors, we averaged the 3-gram vectors in each sequence to form the sequence vectors.

Machine learning model

Machine learning models included Supported Vector Machine (SVM), K-Nearest Neighbor (KNN), Random Forest (RF), Logistic Regression (LR), Decision Tree (DT), Gradient Boosting Decision Tree (GBDT). Naive Bayes (NB) in scikit-learn (https://github.com/scikit-learn/scikit-learn) were recruited. For the basic theory of each adopted model and related literature, we make a brief introduction here.

**LR** measures the relationship between the output and input variables. The logistic regression gives predicted probabilities for each category. This is a relatively simple parametric technique which is widely used in practical applications.

**KNN** is considered as one of the simple and useful predictors for classification task. It works by checking the *k* closest instances in dataset to a new sample, and, making predicting based on which classes the majority of the *k* neighbours belong to (measuring by distance function).

**DT** is built to find decision rules that could be used to conduct prediction from a set of input variables, it has been applied successfully in many real-world situations for classification and prediction (Murthy, 1998).

**RF** is an ensemble method of Decision Trees that are trained separately, the final outcome is estimated by taking results obtained by different trees (estimators) into account.

**NB** classifier is robust to noise and irrelevant attribute, the main advantage of it is that it is simple to construct without any complicated iterative parameter estimation schemes. The prediction $y_{pre}$ could be made by $P(x_{i}/y)$ as follow:

$y_{pre}=argmax P(y)\prod_{i=1}^{n} P(x_{i}/y)$ ()

**SVM** is developed at AT&T Bell Laboratories (Cortes and Vapnik, 1995), which shows good robustness and generalization ability for nonlinear problem. Given the input data $\left\{ \left( x_{i},y_{i} \right), i=1,2,\ldots\ldots,n \right\}$, $x$ is input vector and $y$ is output. The basic idea is to map data into a high dimensional feature space and conduct prediction as follows:

$f\left( x \right)=\sum_{i=1}^{n} \alpha_{i}y_{i}k\left( x_{i},x \right)+b$ ()

Where $k\left( \cdot,\cdot\right)$is kernel function. The performance of SVM depends on parameters including the cost of the error $C$, the width of the loss function $\varepsilon$ and kernel function.

**GBDT** ensembles multiple decision trees by gradient boosting technique to affords strong predictive power. In the classification task, GBDT uses the linear combination of basic estimators to achieve the purpose by continuously reducing the training error. In each iteration, GBDT increases the update value of the previous round of gradient. Therefore, each iteration fits the negative gradient of the loss function in the current state, by which the iterative training process can reduce the loss at the fastest speed. Tuning the value of the number of estimater and concerning learning rate in GBDT is essential process to aviod over-fitting.

During the model training stage, the parameters for SVM, RF, LR, GBDT and KNN were adjusted by grid search, while the parameters of DT and NB were used as default. For SVM, Radial Basis Function (RBF) was used as the kernel function and grid search was done to C and $\varepsilon$. For RF, grid search was done to the number of estimators. For LR, grid search was done to C. For GBDT, grid search was done to learning rate and the number of estimators. For KNN, when weights were set to ‘uniform’, grid search was only done to the number of neighbors; when weights were set to ‘distance’, grid search was done to the number of neighbors and p (distance metric).

Machine learning models involved in this study were evaluated by Accuracy, F1, Precision, Sensitivity, Specificity and Matthews’s correlation coefficient (MCC), concerning index are as follow:

TP (True positive): The number of proteins predicted as PSPs and they were real PSPs.

TN (True negative): The number of proteins predicted as none-PSPs and they were theoretically unlikely to undergo LLPS.

FP (False positive): The number of proteins predicted as PSPs but in fact they were theoretically unlikely to undergo LLPS.

FN (False negative): The number of proteins predicted as none-PSPs but were actually PSPs.

$$A\text{ccu}racy=\frac{TP+TN}{TP+TN+FP+FN}$$

$$F1=\frac{2TP}{2TP+FP+FN}$$

$$\Pr ecision=\frac{TP}{TP+FP}$$

$$Sensitivity=\frac{TP}{TP+FN}$$

$$Specificty=\frac{TN}{TN+FP}$$

$$MCC=\frac{TP\times TN-FP\times FN}{\sqrt{(TP+FP)(TP+FN)(TN+FP)(TN+FN)}}$$

For our best model (Model 1), GBDT model parameters were: n_estimators=200, learning rate=0.1, loss=deviance, alpha=0.9, max_features= None, max_depth=None, subsample=1, min_samples_split=2, min_samples_leaf=1, max_leaf_nodes=None, min_impurity_split=1e-7, presort=FALSE.

Considering that the size of N1 is much larger than P1, we repeated the training process for three times, each time the negative training samples were selected from N1 by random sampling. In the main text, we only presented the results of model 1, the training results of other models and the results of three repeats are shown in Table S1 and Table S2.

PSP/Non-PSP separation threshold setting

Considering the uncertainty within the negative dataset and because most of the first-generation tools only return prediction scores, it was necessary to set a reasonable PSP/Non-PSP separation threshold. Previous tools have set this threshold using the whole proteome as a background, for example, top 1.8% (Vernon and Forman-Kay, 2019), 2% (Youn et al., 2019) and 20% (Vernon and Forman-Kay, 2019), scored proteins in whole proteome were regarded as PSPs and were regarded as Non-PSPs if not. In this work, for the positive dataset T1+, we used a recall-receive curve to analyze the recalls under different threshold of top *n* percent of the proteome scores (*n* was between 0 and 25%). For Youn’s dataset and DrLLPS, we set the threshold to 2%, which was the same as Youn’s et al. For model training accuracy calculation and IDP prediction, score of 0.5 (instead of a background threshold) was used as a direct threshold. For human proteome scan, in order to identify most likely PSPs, we set the threshold to 1.8%.

Human PSPs GO terms enrichment analysis

Human proteins with scores in the top 1.8% of human proteome were uploaded to DAVID 6.8 (https://david.ncifcrf.gov/)(Huang et al., 2007). We analyzed human PSPs for the enrichment of GO terms for cellular components, and each term received a p-value (EASE score). The resulting GO terms were compared with another report (Vernon and Forman-Kay, 2019), which presented GO terms that were enriched in the PSPs predicted by first-generation tools. Then, we analyzed annotation clusters for GO terms associated with cellular components, biologic processes, and molecular functions. For terms with EASE scores <0.1, we calculated the geometric mean of EASE scores with similar biologic meanings to determine the enrichment score for each cluster. GO term clusters with high enrichment scores were the most related functions of PSPs predicted by PSPredictor.

Sequence redundancy removal

We removed sequence redundancy so that no two sequences in the dataset had a similarity of ≥50%. We used the CD-hit (Li and Godzik, 2006) (http://weizhongli-lab.org/cd-hit/) remote package to remove redundancy by sequence clustering. The represented sequences in each cluster were selected to form the redundancy removal datasets.

Sequence comparison

NCBI blast+ 2.2.31(Camacho et al., 2009) remote package was used to compare pairs of protein sequences.

Sequence domain annotation

Hmmer 3.1b2 (Potter et al., 2018) (http://hmmer.org/publications.html) remote package was used to annotate sequence domains.

Data and Code Availability

The training datasets are available at GitHub (<https://github.com/pkumdl/PSPredict>or).

References

Burley, S.K., Berman, H.M., Bhikadiya, C., Bi, C., Chen, L., Di Costanzo, L., Christie, C., Dalenberg, K., Duarte, J.M., Dutta, S.*, et al.* (2018). RCSB Protein Data Bank: biological macromolecular structures enabling research and education in fundamental biology, biomedicine, biotechnology and energy. Nucleic Acids Res *47*, D464-D474.

Camacho, C., Coulouris, G., Avagyan, V., Ma, N., Papadopoulos, J., Bealer, K., and Madden, T.L. (2009). BLAST+: architecture and applications. BMC Bioinformatics *10*, 421.

Cortes C., Vapnik V.N. (1995), “Support Vector Networks”, Machine Learning, Vol.20, pp.273-297.

Huang, D.W., Sherman, B.T., Tan, Q., Kir, J., Liu, D., Bryant, D., Guo, Y., Stephens, R., Baseler, M.W., and Lane, H.C. (2007). DAVID Bioinformatics Resources: expanded annotation database and novel algorithms to better extract biology from large gene lists. Nucleic Acids Res *35*, W169-W175.

Li, Q.L., and Lai, L.H. (2007). Prediction of potential drug targets based on simple sequence properties. BMC Bioinformatics *8*, 1-11.

Li, W., and Godzik, A. (2006). Cd-hit: a fast program for clustering and comparing large sets of protein or nucleotide sequences. Bioinformatics *22*, 1658-1659.

Mikolov, T., Sutskever, I., Kai, C., Corrado, G., and Dean, J. (2013). Distributed representations of words and phrases and their compositionality. Adv Neural Inf Process Syst *26*, 3111-3119.

Murthy, S.K. (1998). Automatic construction of decision trees from data: a multi-disciplinary survey. Data Mining and Knowledge Discovery 2 (4), 345–389.

Ning, W.S., Guo, Y.P., Lin, S.F., Mei, B., Wu, Y., Jiang, P.R., Tan, X.D., Zhang, W.Z., Chen, G.W., Peng, D.*, et al.* (2019). DrLLPS: a data resource of liquid–liquid phase separation in eukaryotes. Nucleic Acids Res.

Potter, S.C., Luciani, A., Eddy, S.R., Park, Y., Lopez, R., and Finn, R.D. (2018). HMMER web server: 2018 update. Nucleic Acids Res *46*, W200-W204.

Sun, T.L., Lai, L.H., and Pei, J.F. (2018). Analysis of protein features and machine learning algorithms for prediction of druggable proteins. Quantitative Biology *6*.

Sun, T.L., Zhou, B., Lai, L.H., and Pei, J.F. (2017). Sequence-based prediction of protein protein interaction using a deep-learning algorithm. BMC Bioinformatics *18*, 277.

Vaswani, A., Shazeer, N., Parmar, N., et al. (2017). Attention is all you need. Advances in Neural Information Processing Systems 5998-6008.

Vernon, R.M., and Forman-Kay, J.D. (2019). First-generation predictors of biological protein phase separation. Curr Opin Struct Biol *58*, 88-96.

Youn, J.-Y., Dyakov, B.J.A., Zhang, J.P., Knight, J.D.R., Vernon, R.M., Forman-Kay, J.D., and Gingras, A.-C. (2019). Properties of Stress Granule and P-Body Proteomes. Mol Cell *76*, 286-294.
