# Supplementary material for: Prediction of liquid–liquid phase separating proteins using machine learning
Source: BMC Bioinformatics. 2022 Feb 15;23:72. doi: 10.1186/s12859-022-04599-w (PMC8845408; doi:10.1186/s12859-022-04599-w)
Supplement: Supplementary file 2 — Additional file 2: Table S1. All models’ training results; Table S2. The training results of three repeats of models with (1) w2v coded, (2) the ratio of positive samples and negative samples is 1:1, (3) sequence number is 586, and, (4) GBDT trained. [file 12859_2022_4599_MOESM2_ESM.docx]

# Appendix

**Table S 1.** All models’ training results.

| Protein coding | Ratio | Sample number | Algorithms | Accuracy^a^ | F1^a^ | Precision^a^ | Sensitivity^a^ | Specificty^a^ | MCC^a^ |
| --- | --- | --- | --- | --- | --- | --- | --- | --- | --- |
| w2v | 1 | 586 | NB | 0.6103 | 0.5910 | 0.6161 | 0.5721 | 0.6485 | 0.2221 |
|  |  |  | KNN | 0.8563 | 0.8296 | 0.9168 | 0.7641 | 0.9284 | 0.7058 |
|  |  |  | LR | 0.9248 | 0.9220 | 0.9535 | 0.8941 | 0.9555 | 0.8526 |
|  |  |  | RF | 0.8939 | 0.8925 | 0.9041 | 0.8836 | 0.9043 | 0.7900 |
|  |  |  | DT | 0.8652 | 0.8616 | 0.8791 | 0.8463 | 0.8841 | 0.7321 |
|  |  |  | SVM | 0.7679 | 0.7465 | 0.8182 | 0.6891 | 0.8467 | 0.5437 |
|  |  |  | GBDT | 0.9471 | 0.9466 | 0.9612 | 0.9352 | 0.9591 | 0.8970 |
| LQL | 1 | 586 | NB | 0.6676 | 0.6229 | 0.7202 | 0.5532 | 0.7821 | 0.3465 |
|  |  |  | KNN | 0.9101 | 0.9050 | 0.9520 | 0.8641 | 0.9560 | 0.8248 |
|  |  |  | LR | 0.9167 | 0.9123 | 0.9592 | 0.8739 | 0.9594 | 0.8397 |
|  |  |  | RF | 0.9216 | 0.9195 | 0.9429 | 0.9009 | 0.8473 | 0.8830 |
|  |  |  | DT | 0.8988 | 0.8477 | 0.8552 | 0.8433 | 0.7736 | 0.8909 |
|  |  |  | SVM | 0.9421 | 0.9405 | 0.9674 | 0.9180 | 0.9662 | 0.8880 |
|  |  |  | GBDT | 0.8909 | 0.8907 | 0.8969 | 0.8872 | 0.8946 | 0.8966 |
| w2v | 2 | 879 | NB | 0.6885 | 0.4670 | 0.4138 | 0.5393 | 0.7383 | 0.2584 |
|  |  |  | KNN | 0.8737 | 0.7783 | 0.9317 | 0.6722 | 0.9744 | 0.7134 |
|  |  |  | LR | 0.9340 | 0.8927 | 0.9630 | 0.8363 | 0.9829 | 0.8522 |
|  |  |  | RF | 0.9178 | 0.8752 | 0.8986 | 0.8563 | 0.9486 | 0.8163 |
|  |  |  | DT | 0.9010 | 0.8512 | 0.8532 | 0.8531 | 0.9248 | 0.7790 |
|  |  |  | SVM | 0.7929 | 0.5967 | 0.8630 | 0.4614 | 0.9589 | 0.5189 |
|  |  |  | GBDT | 0.9476 | 0.9187 | 0.9433 | 0.9007 | 0.9709 | 0.8833 |
| LQL | 2 | 879 | NB | 0.7405 | 0.5111 | 0.6931 | 0.4126 | 0.9044 | 0.3758 |
|  |  |  | KNN | 0.9192 | 0.8673 | 0.9517 | 0.7986 | 0.9795 | 0.8174 |
|  |  |  | LR | 0.9125 | 0.8487 | 0.9691 | 0.7647 | 0.9864 | 0.8047 |
|  |  |  | RF | 0.9136 | 0.8591 | 0.9273 | 0.8091 | 0.9658 | 0.8056 |
|  |  |  | DT | 0.8988 | 0.8477 | 0.8552 | 0.8433 | 0.9266 | 0.7736 |
|  |  |  | SVM | 0.9534 | 0.9253 | 0.9787 | 0.8806 | 0.9898 | 0.8959 |
|  |  |  | GBDT | 0.9420 | 0.9084 | 0.9505 | 0.8738 | 0.9761 | 0.8698 |
| w2v | 5 | 1758 | NB | 0.7372 | 0.3790 | 0.3138 | 0.4813 | 0.7884 | 0.2307 |
|  |  |  | KNN | 0.9284 | 0.7327 | 0.9534 | 0.6015 | 0.9939 | 0.7216 |
|  |  |  | LR | 0.9511 | 0.8297 | 0.9764 | 0.7241 | 0.9966 | 0.8154 |
|  |  |  | RF | 0.9465 | 0.8244 | 0.9052 | 0.7613 | 0.9836 | 0.7991 |
|  |  |  | DT | 0.9380 | 0.8042 | 0.8459 | 0.7721 | 0.9713 | 0.7708 |
|  |  |  | SVM | 0.8453 | 0.1305 | 0.8000 | 0.0720 | 0.9612 | 0.2695 |
|  |  |  | GBDT | 0.9625 | 0.8793 | 0.9470 | 0.8229 | 0.9904 | 0.8611 |
| LQL | 5 | 1758 | NB | 0.8441 | 0.3514 | 0.5639 | 0.2592 | 0.9611 | 0.5639 |
|  |  |  | KNN | 0.9494 | 0.8275 | 0.9529 | 0.7339 | 0.9925 | 0.8089 |
|  |  |  | LR | 0.9437 | 0.8013 | 0.9673 | 0.6860 | 0.9952 | 0.7859 |
|  |  |  | RF | 0.9670 | 0.8913 | 0.9716 | 0.8254 | 0.9952 | 0.8769 |
|  |  |  | DT | 0.9318 | 0.7970 | 0.7972 | 0.8018 | 0.9577 | 0.7579 |
|  |  |  | SVM | 0.9727 | 0.9143 | 0.9612 | 0.8736 | 0.9925 | 0.9002 |
|  |  |  | GBDT | 0.9699 | 0.9042 | 0.9546 | 0.8603 | 0.9918 | 0.8886 |
| a. The standard deviation of these indexes is between 0.010-0.050 | | | | | | | | | |

**Table S 2.** The training results of three repeats of models with (1) w2v coded, (2) the ratio of positive samples and negative samples is 1:1, (3) sequence number is 586, and, (4) GBDT trained.

|  | Accuracy^a^ | F1^a^ | Precision^a^ | Sensitivity^a^ | Specificty^a^ | MCC^a^ |
| --- | --- | --- | --- | --- | --- | --- |
| 1 | 0.9471 | 0.9466 | 0.9612 | 0.9352 | 0.9591 | 0.8970 |
| 2 | 0.9455 | 0.9445 | 0.9560 | 0.9353 | 0.9557 | 0.8932 |
| 3 | 0.9335 | 0.9322 | 0.9455 | 0.9213 | 0.9457 | 0.8691 |
| a. The standard deviation of these indexes is between 0.010-0.050 | | | | | | |
